# The effects of progesterone on the alpha2-adrenergic receptor subtypes in late-pregnant uterine contractions in vitro

**DOI:** 10.1186/s12958-016-0166-9

**Published:** 2016-06-14

**Authors:** Judit Hajagos-Tóth, Judit Bóta, Eszter Ducza, Reza Samavati, Anna Borsodi, Sándor Benyhe, Róbert Gáspár

**Affiliations:** Department of Pharmacodynamics and Biopharmacy, Faculty of Pharmacy, University of Szeged, Szeged, H-6701 P.O. Box 121, Hungary; Institute of Biochemistry, Biological Research Centre, Hungarian Academy of Sciences, Temesvari krt 62, Szeged, H-6726 Hungary

**Keywords:** Progesterone, Gestation, Rat, Myometrium, α_2_-adrenergic receptor subtypes

## Abstract

**Background:**

The adrenergic system and progesterone play major roles in the control of the uterine function. Our aims were to clarify the changes in function and expression of the α_2_-adrenergic receptor (AR) subtypes after progesterone pretreatment in late pregnancy.

**Methods:**

Sprague Dawley rats from pregnancy day 15 were treated with progesterone for 7 days. The myometrial expressions of the α_2_-AR subtypes were determined by RT-PCR and Western blot analysis. In vitro contractions were stimulated with (−)-noradrenaline, and its effect was modified with the selective antagonists BRL 44408 (α_2A_), ARC 239 (α_2B/C_) and spiroxatrine (α_2A_). The accumulation of myometrial cAMP was also measured. The activated G-protein level was investigated via GTPγS binding assays.

**Results:**

Progesterone pretreatment decreased the contractile effect of (−)-noradrenaline through the α_2_-ARs. The most significant reduction was found through the α_2B_-ARs. The mRNA of all of the α_2_-AR subtypes was increased. Progesterone pretreatment increased the myometrial cAMP level in the presence of BRL 44408 (*p* < 0.001), spiroxatrine (*p* < 0.001) or the spiroxatrine + BRL 44408 combination (*p* < 0.05). Progesterone pretreatment increased the G-protein-activating effect of (−)-noradrenaline in the presence of the spiroxatrine + BRL 44408 combination.

**Conclusions:**

The expression of the α_2_-AR subtypes is progesterone-sensitive. It decreases the contractile response of (−)-noradrenaline through the α_2B_-AR subtype, blocks the function of α_2A_-AR subtype and alters the G protein coupling of these receptors, promoting a G_s_-dependent pathway. A combination of α_2C_-AR agonists and α_2B_-AR antagonists with progesterone could be considered for the treatment or prevention of preterm birth.

## Background

The physiology of uterine quiescence and contractility is very complex. Myometrial contraction is regulated by a number of factors, such as female sexual hormones, the adrenergic receptor (AR) system, ion channels and transmitters. However, the exact cellular and molecular events are still in question. Dysregulation of the myometrial contractility can lead to either preterm or slow-to-progress labor. It is therefore crucial to understand the mechanisms that regulate uterine contractility in order to prevent or treat the pathological processes related to the pregnant myometrium [1–4].

It is well known that the female sexual hormone progesterone is responsible for uterine quiescence [5, 6], while estrogens have major role in myometrial contractions [1, 7]. The progesterone level normally declines at term prior to the development of labor and it is therefore used to prevent threatening preterm birth [8, 9]. Progesterone and estrogen also play an important role in the regulation of the adrenergic system [10]. Estrogen decreases the expressions of the α_2_-AR subtypes and alters the myometrial contracting effect of (−)-noradrenaline by reduced coupling of the α_2B_-ARs to G_i_ protein [11]. Progesterone enhances the synthesis of β_2_-ARs during gestation [12–14], and the number of activated G-proteins [12, 15], and β_2_-AR agonists can therefore theoretically be combined with progesterone in threatening premature labour [16]. The myometrial α_1_-AR is also influenced by progesterone. It induces a change in the G_q_/G_i_-activating property of the α_1AD_-AR in rats [17]. However, the effect of progesterone on the myometrial α_2_-AR subtypes is still unknown. Since progesterone has a major role in myometrial quiescence during human parturition [18], it seems important to know its direct influence on the α_2_-AR subtypes, which are also involved in the mechanism of uterine contractions [19].

The α_2_-ARs have been divided into three groups [20, 21], the α_2A,_ α_2B_ and α_2C_ subtypes. All of three receptor subtypes bind to the pertussis toxin-sensitive G_i_ protein [22] and decreases the activity of adenylyl cyclase (AC) [23], but under certain circumstances α_2_-ARs can also couple to G_s_-proteins and increase adenylyl cyclase activity [24]. All three receptor subtypes are involved in various physiological functions, and especially in the cardiovascular and central nervous systems [25]. Furthermore, all of them have been identified in both the pregnant and the non-pregnant myometrium, and have been shown to take part in both increased and decreased myometrial contractions [26]. The α_2B_-ARs predominate and mediate contraction at the end of gestation in rats, decreasing the intracellular cAMP level, while the stimulation of the myometrial α_2A_- and α_2C_-ARs increases the cAMP level, and mediates only weak contractions [27].

Since no data are available on the effects of progesterone on the myometrial functions of the different α_2_-AR subtypes, we set out to clarify the changes in expression and function of the α_2A_-, α_2B_- and α_2C_-AR subtypes after progesterone pretreatment on the last day of pregnancy in rats.

## Methods

The animal experimentation was carried out with the approval of the Hungarian Ethical Committee for Animal Research (permission number: IV/198/2013). The animals were treated in accordance with the EU Directive 2010/63/EU for animal experiments and the Hungarian Act for the Protection of Animals in Research (XXVIII. tv. 32.§).

### Housing and handling of the animals

Sprague–Dawley rats were obtained from the INNOVO Ltd. (Gödöllő, Hungary) and were housed under controlled temperature (20–23 °C), in humidity (40–60%) and light (12 h light/dark regime)-regulated rooms. The animals were kept on a standard rodent pellet diet (INNOVO Ltd., Isaszeg, Hungary), with tap water available *ad libitum*.

### Mating of the animals

Mature female (180–200 g) and male (240–260 g) Sprague–Dawley rats were mated in a special mating cage in the early morning hours. A time-controlled metal door separated the rooms for the male and female animals. The separating door was opened before dawn (4 a.m.) Within 4–5 h after the possibility of mating, intercourse was confirmed by the presence of a copulation plug or vaginal smears. In positive cases, the female rats were separated and this was regarded as the first day of pregnancy.

### In vivo sexual hormone treatments of the rats

The progesterone (Sigma-Aldrich, Budapest, Hungary) pretreatment of the pregnant animals was started on day 15 of pregnancy. Progesterone was dissolved in olive oil and injected subcutaneously every day up to day 21 in a dose of 0.5 mg/0.1 ml [28].

On day 22, the uterine samples were collected, and contractility and molecular pharmacological studies were carried out.

### RT-PCR studies

Tissue isolation: Rats (250–350 g) were sacrificed by CO_2_ asphyxiation. Fetuses were sacrificed by immediate cervical dislocation. The uterine tissues from pregnant animals (tissue between two implantation sites) were rapidly removed and placed in RNAlater Solution (Sigma-Aldrich, Budapest, Hungary). The tissues were frozen in liquid nitrogen and then stored at −70 °C until the extraction of total RNA.

Total RNA preparation from tissue: Total cellular RNA was isolated by extraction with guanidinium thiocyanate-acid-phenol-chloroform according to the procedure of Chomczynski and Sacchi [29]. After precipitation with isopropanol, the RNA was washed with 75% ethanol and then re-suspended in diethyl pyrocarbonate-treated water. RNA purity was controlled at an optical density of 260/280 nm with BioSpec Nano (Shimadzu, Japan); all samples exhibited an absorbance ratio in the range 1.6–2.0. RNA quality and integrity were assessed by agarose gel electrophoresis.

Reverse transcription and amplification of the PCR products was performed by using the TaqMan RNA-to-CTTM 1-Step Kit (Life Technologies, Budapest, Hungary) and the ABI StepOne Real-Time cycler. RT-PCR amplifications were performed as follows: 48 °C for 15 min and 95 °C for 10 min, followed by 40 cycles at 95 °C for 15 s and 60 °C for 1 min. The generation of specific PCR products was confirmed by melting curve analysis. Table 1 contains the assay IDs for the used primers. The amplification of β-actin served as an internal control. All samples were run in triplicates. The fluorescence intensities of the probes were plotted against PCR cycle numbers. The amplification cycle displaying the first significant increase in the fluorescence signal was defined as the threshold cycle (CT).

**Table 1:** Parameters of the applied primers and PCR reactions. The real-time reverse transcription polymerase chain reactions were used to determine the changes in the mRNA expression. In our studies the parameters of inventoried TaqMan assays were defined by Life Technologies (ThermoFisher Scientific, Hungary)

TaqMan assays	Assay ID (Life Technologies, Hungary)	Accession number	Assay location	Amplicon length	Annealing temp. (°C)	Reaction volume (μ*l*)
α_2A_-AR	Rn00562488_s1	NM_012739.3	1350	72	60	20
α_2B_-AR	Rn00593312_s1	NM_138505.2	1451	63	60	20
α_2C_-AR	Rn00593341_s1	NM_138506.1	653	111	60	20
β-actin	Rn00667869_m1	NM_031144.3	881	91	60	20

### Western blot analysis

Twenty μg of protein per well was subjected to electrophoresis on 4–12% NuPAGE Bis-Tris Gel in XCell SureLock Mini-Cell Units (Life Technologies, Budapest, Hungary). Proteins were transferred from gels to nitrocellulose membranes, using the iBlot Gel Transfer System (Life Technologies, Hungary). The antibody binding was detected with the WesternBreeze Chromogenic Western blot immundetection kit (Life Technologies, Budapest, Hungary). The blots were incubated on a shaker with α_2A_-AR, α_2B_-AR, α_2C_-AR and β-actin polyclonal antibody (Santa Cruz Biotechnology, California, 1:200) in the blocking buffer. Images were captured with the EDAS290 imaging system (Csertex Ltd., Hungary), and the optical density of each immunoreactive band was determined with Kodak 1D Images analysis software. Optical densities were calculated as arbitrary units after local area background subtraction.

### Isolated organ studies

Uteri were removed from the 22-day-pregnant rats (250–350 g). 5 mm-long muscle rings were sliced from both horns of the uterus and mounted vertically in an organ bath containing 10 ml de Jongh solution (composition: 137 mM NaCl, 3 mM KCl, 1 mM CaCl_2_, 1 mM MgCl_2_, 12 mM NaHCO_3_, 4 mM NaH_2_PO_4_, 6 mM glucose, pH = 7.4). The temperature of the organ bath was maintained at 37 °C, and carbogen (95% O_2_ + 5% CO_2_) was perfused through the bath. After mounting, the rings were allowed to equilibrate for approximately 60 min before experiments were started, with a buffer change every 15 min. The initial tension of the preparation was set to about 1.5 g and the tension dropped to about 0.5 g by the end of the equilibration period. The tension of the myometrial rings was measured with a gauge transducer (SG-02; Experimetria Ltd., Budapest, Hungary) and recorded with a SPEL Advanced ISOSYS Data Acquisition System (Experimetria Ltd., Budapest, Hungary). In the following step contractions were elicited with (−)-noradrenaline (10^−8^ to 10^-4.5^ M) and cumulative concentration–response curves were constructed in each experiment in the presence of doxazosin (10^−7^ M) and propranolol (10^−5^ M) in order to avoid α_1_-adrenergic and β-adrenergic actions. Selective α_2_-AR subtype antagonists (each 10^−7^ M), propranolol and doxazosin were left to incubate for 20 min before the administration of contracting agents. Following the addition of each concentration of (−)-noradrenaline, recording was performed for 300 s. Concentration–response curves were fitted and areas under curves (AUC) were evaluated and analysed statistically with the Prism 4.0 (Graphpad Software Inc. San Diego, CA, USA) computer program. From the AUC values, E_max_ and EC_50_ values were calculated. Statistical evaluations were carried out with the ANOVA Dunnett test or the two-tailed unpaired t-test.

### Measurement of uterine cAMP accumulation

Uterine cAMP accumulation was measured with a commercial cAMP Enzyme Immunoassay Kit (Cayman Chemical, USA). Uterine tissue samples (control and 17β-estradiol-treated) from 22-day-pregnant rats were incubated in an organ bath (10 ml) containing de Jongh solution (37 °C, perfused with carbogen). Isobutylmethylxanthine (10^−3^ M), doxazosin (10^−7^ M), propranolol (10^−5^ M) and the investigated subtype-selective α_2_-AR antagonists (each 10^−7^ M) were incubated with the tissues for 20 min, and (−)-noradrenaline (3 × 10^−6^ M) were added to the bath for 10 min. At the end of the (−)-noradrenaline incubation period, forskolin (10^−5^ M) was added for another 10 min. After stimulation, the samples were immediately frozen in liquid nitrogen and stored until the extraction of cAMP [30]. Frozen tissue samples were then ground, weighed, homogenized in 10 volumes of ice-cold 5% trichloroacetic acid and centrifuged at 1000*g* for 10 min. The supernatants were extracted with 3 volumes of water-saturated diethyl ether. After drying, the extracts were stored at −70 °C until the cAMP assay. Uterine cAMP accumulation was measured with a commercial competitive cAMP EIA Kit; tissue cAMP levels were expressed in pmol/mg tissue.

### [^35^S]GTPγS binding assay

Uteri were removed and homogenized in 20 volumes (w/v) of ice-cold buffer (10 mM Tris–HCl, 1 mM EDTA, 0.6 mM MgCl_2_, and 0.25 M sucrose, pH 7.4) with an Ultra Turret T25 (Janke & Kunkel, Staufen, Germany) homogenizer, and the suspension was then filtered on four layers of gauze and centrifuged (40,000*g*, 4 °C, 20 min). After centrifugation, the pellet was resuspended in a 5-fold volume of buffer. The protein contents of the samples were diluted to 10 mg protein/sample. Membrane fractions was incubated in a final volume of 1 ml at 30 °C for 60 min in Tris-EGTA buffer (pH 7.4) composed of 50 mM Tris–HCl, 1 mM EGTA, 3 mM MgCl_2_, 100 mM NaCl, containing 20 MBq/0.05 cm^3^ [^35^S]GTPγS (0.05 nM) (Sigma Aldrich, Budapest, Hungary), together with increasing concentrations (10^−9^–10^−5^ M) of (−)-noradrenaline. BRL 44408, ARC 239 and spiroxatrine were used in a fixed concentration of 0.1 μM. For the blocking of α_1_- and β-ARs, doxazosin and propranolol were used in a fixed concentration of 10 μM. Total binding was measured in the absence of the ligands, non-specific binding was determined in the presence of 10 μM unlabeled GTPγS and subtracted from total binding. The difference represents basal activity. Bound and free [^35^S]GTPγS were separated by vacuum filtration through Whatman GF/B filters with Brandel M24R Cell harvester. Filters were washed three times with 5 ml ice-cold buffer (pH 7.4), and the radioactivity of the dried filters was detected in UltimaGold™ MV scintillation cocktail with Packard Tricarb 2300TR liquid scintillation counter [31]. The [^35^S]GTPγS binding experiments were performed in triplicate and repeated at least three times.

G_i_ protein was inhibited with pertussis toxin (Sigma Aldrich, Budapest, Hungary) in a concentration of 500 ng/ml after the addition of protein and GDP to the Tris-EGTA buffer 30 min before [^35^S]GTPγS.

## Results

### RT-PCR and Western blot studies

**Fig. 1:**
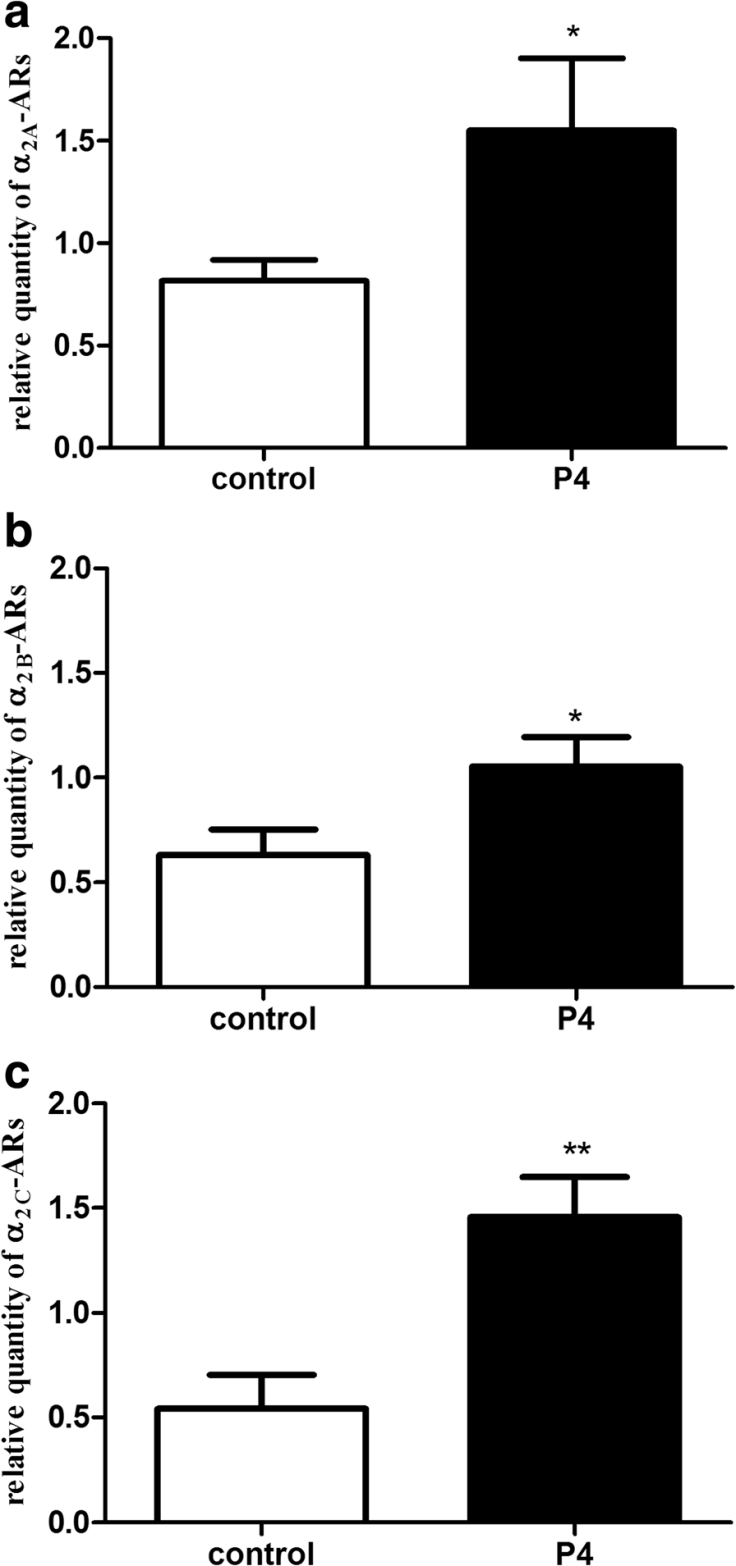
Changes in the myometrial mRNA and protein expression of the α_2A_- (**a**), α_2B_- (**b**) and α_2C_-adrenergic receptors (**c**) after progesterone pretreatment. The statistical analyses were carried out with the two-tailed unpaired t-test. ^*^ *p* < 0.05 ; ^**^ *p* < 0.01

**Fig. 2:**
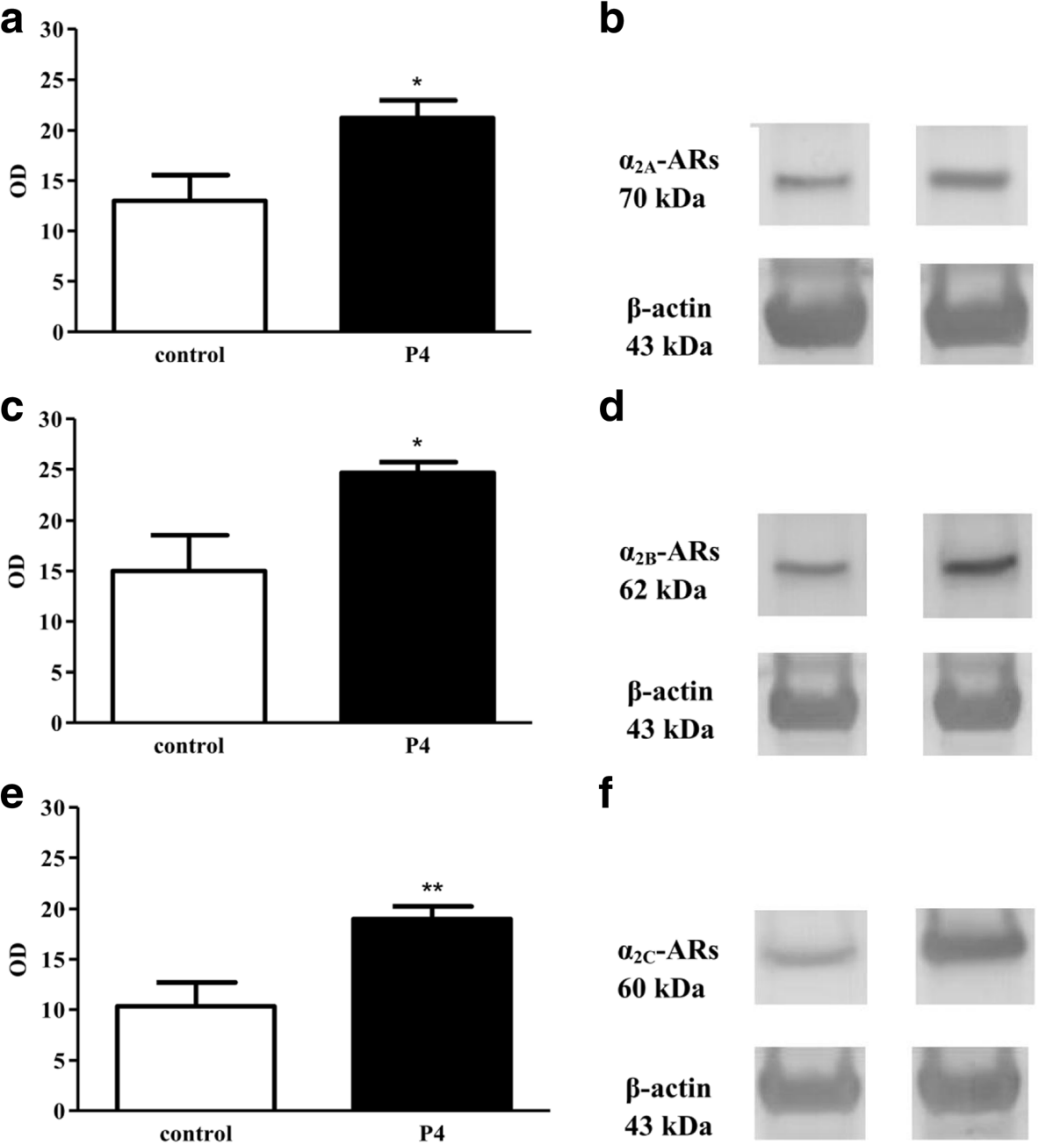
Changes in the α_2_-adrenergic receptor levels in the 22-day pregnant rat myometrium after progesterone pretreatment. The α_2_-adrenergic receptor and β-actin Western blot products for α_2A_- (**b**), α_2B_- (**d**) and α_2C_-adrenergic receptors (**f**). The 70, 62 and 60 kDa proteins relate to the α_2AR_-, α_2B_- and α_2C_- adrenergic receptors and β-actin, respectively. The antibody binding was expressed as optical density (OD) data (**a**) for α_2A_-_,_ (**c**) for α_2B_-and (**e**) for α_2C_-adrenergic receptors. The y axis shows the ratio of α_2_-adrenergic receptors/β-actin protein optical densities. The statistical analyses were carried out with the two-tailed unpaired t-test. ^*^ *p* < 0.05; ^**^ *p* < 0.01

The mRNA expression of each α_2_-AR subtype (Fig. 1a-c) was significantly increased after progesterone pretreatment as compared with the non-treated uteri (*p* < 0.05). The results of Western blot analysis at the level of protein expression revealed a significant increase in each α_2_-AR subtype, which correlated with the PCR results (Fig. 2a-f).

### Isolated organ studies

**Fig. 3:**
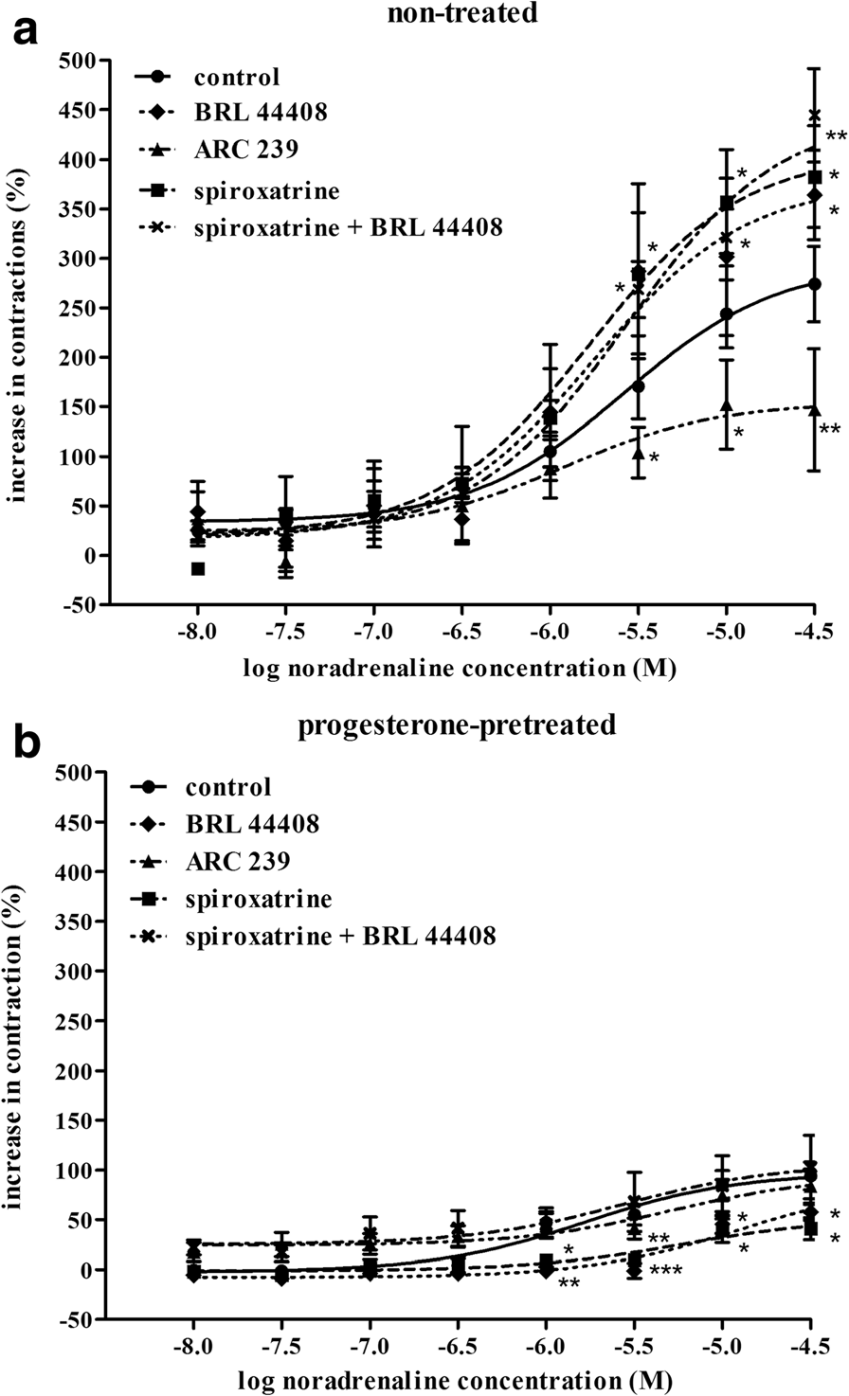
Effects of the subtype-selective α_2A_-adrenergic receptor antagonist BRL 44408, the α_2B/C_-adrenergic receptor antagonist ARC 239 and the α_2C_-adrenergic receptor antagonist spiroxatrine on the (−)-noradrenaline-evoked contractions in the 22-day-pregnant rat myometrium (**a**) and after progesterone pretreatment (**b**). The studies were carried out in the presence of the β-adrenergic receptor antagonist propranolol (10^−5^ M) and the α_1_-adrenergic receptor antagonist doxazosin (10^−7^ M) in each case. The change in contraction was calculated via the area under the curve and expressed in % ± S.E.M. The statistical analyses were carried out with the ANOVA Dunnett test. *p < 0.05; **p < 0.01; ***p < 0.001

In the 22-day-pregnant myometrium, (−)-noradrenaline in the concentration range of 10^−8^ to 10^-4.5^ M increased the myometrial contractions (E_max_ = 274.1 ± 47.1) (Fig. 3a). After progesterone pretreatment, the myometrial contracting effect of (−)-noradrenaline was decreased (E_max_ = 94.0 ± 14.4; *p* < 0.01) (Fig. 3b). The EC_50_ and E_max_ values of the curves are listed in Table 2a.

**Table 2:** Changes in the uterus-contracting effect of (−)-noradrenaline (EC_50_ and E_max_ values) in the absence of α_2_-antagonists (a), or in the presence of an α_2A_-antagonist (b), an α_2B/C_-antagonist (c), an α_2C_-antagonist (d) or an α_2A_- and an α_2C_-antagonists (e) in the 22-day-pregnant rat without and after progesterone pretreatment

	EC_50_ (M ± S.E.M.)	E_max_ (% ± S.E.M)
(a) CONTROL		
Non-treated	2.6×10^−6^ ± 1.2×10^−6^	274.1 ± 47.1
Progesterone-pretreated	1.7×10^−6^ ± 7.8×10^−7 ns^	94.0 ± 14.4 **
(b) BRL 44408		
Non-treated	1.8×10^−6^ ± 6.4×10^−6^	364.3 ± 70.8
Progesterone-pretreated	1.2×10^−5^ ± 4.8 ×10^−6 ns^	57.4 ± 12.9 ***
(c) ARC 239		
Non-treated	1.2×10^−6^ ± 1.1×10^−6^	147.1 ± 79.4
Progesterone-pretreated	5.3×10^−6^ ± 1.5×10^−6 ns^	83.7 ± 22.1*
(d) SPIROXATRINE		
Non-treated	1.6×10^−6^ ± 5.4×10^−6^	382.4 ± 93.5
Progesterone-pretreated	5.9×10^−6^ ± 1.4×10^−6 ns^	41.0 ± 15.2 ***
(e) BRL 44408 + SPIROXATRINE		
Non-treated	2.9×10^−6^ ± 8.4×10^−7^	444.6 ± 51.3
Progesterone-pretreated	2.8×10^−6^ ± 2.7 ×10^−5 ns^	102.9 ± 32.62 ***

EC_50_: the concentration of (−)-noradrenaline alone or in the presence of an α_2_-AR antagonist which elicits half of the maximum contracting effect of (−)-noradrenaline. E_max_: the maximum contracting effect of (−)-noradrenaline alone or in the presence of an α_2_-AR antagonist. Significance levels were calculated in comparison with non-treated values. ns: not significant; *: *p* < 0.05; **: *p* < 0.01; ***: *p* < 0.001

In the presence of the α_2A_-AR antagonist BRL 44408, progesterone pretreatment decreased the (−)-noradrenaline-evoked contractions as compared with the progesterone-treated control (*p* < 0.05) (Fig. 3b). BRL 44408 enhanced the (−)-noradrenaline-induced contractions, this being markedly reduced by progesterone pretreatment (*p* < 0.001) (Fig. 3a, b; Table 2b).

In the presence of the α_2B/C_-AR antagonist ARC 239, progesterone pretreatment did not modify the myometrial contracting effect of (−)-noradrenaline relative to the progesterone-treated control. The concentration-response curve was very flat, the difference between the minimum and the maximum effect was less then 20% (Fig. 3b). ARC 239 reduced the (−)-noradrenaline-induced contractions, which were decreased further by progesterone pretreatment (*p* < 0.05) (Fig. 3a, b; Table 2c).

Progesterone pretreatment decreased the maximum contracting effect of (−)-noradrenaline in the presence of spiroxatrine as compared with the progesterone-treated control (*p* < 0.05) (Fig. 3b). Spiroxatrine enhanced the (−)-noradrenaline-induced contractions, which were enormously reduced by progesterone pretreatment (*p* < 0.001) (Fig. 3a, b; Table 2d).

In the presence of the combination of spiroxatrine + BRL 44408, progesterone pretreatment did not modify the maximum myometrial contracting effect of (−)-noradrenaline in comparison with the progesterone-treated control (Fig. 3b). The combination of the two compounds increased the (−)-noradrenaline-induced contractions, which were reduced by progesterone pretreatment (*p* < 0.001) (Fig. 3a, b; Table 2e).

### cAMP studies

**Fig. 4:**
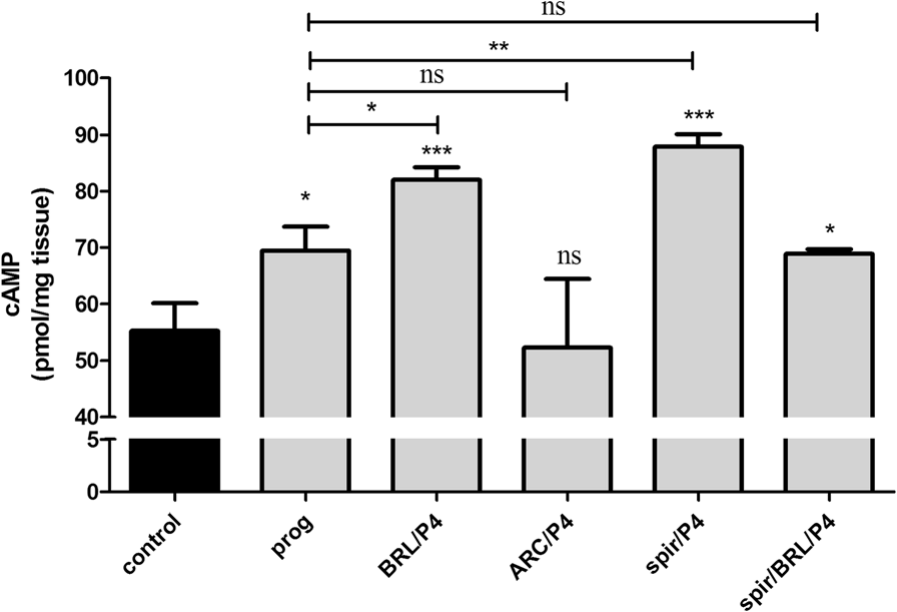
Effects of the subtype-selective α_2A_-adrenergic receptor antagonist BRL 44408, the α_2B/C_-adrenergic receptor antagonist ARC 239 and the α_2C_-adrenergic receptor antagonist spiroxatrine on the myometrial cAMP level (pmol/mg tissue ± S.D.) in the presence of isobutylmethylxanthine (10^−3^ M) and forskolin (10^−5^ M) (control) in the 22-day-pregnant rat (*n* = 6) after progesterone pretreatment. The statistical analyses were carried out with ANOVA followed by Dunnett’s Multiple Comparison Test. *p < 0.05; **p < 0.01; ***p < 0.001

Progesterone pretreatment increased the myometrial cAMP level (*p* < 0.05) (Fig. 4) produced in the presence of (−)-noradrenaline. The myometrial cAMP level was also increased in the presence of BRL 44408 (*p* < 0.001), spiroxatrine (*p* < 0.001) and the spiroxatrine + BRL 44408 combination (*p* < 0.05). However, ARC 239 did not modify the amount of myometrial cAMP level after progesterone pretreatment. In addition, BRL 44408 (*p* < 0.05) and spiroxatrine (*p* < 0.01) increased the myometrial cAMP level compared to the progesterone-treated control.

### [^35^S]GTPγS binding assay studies

**Fig. 5:**
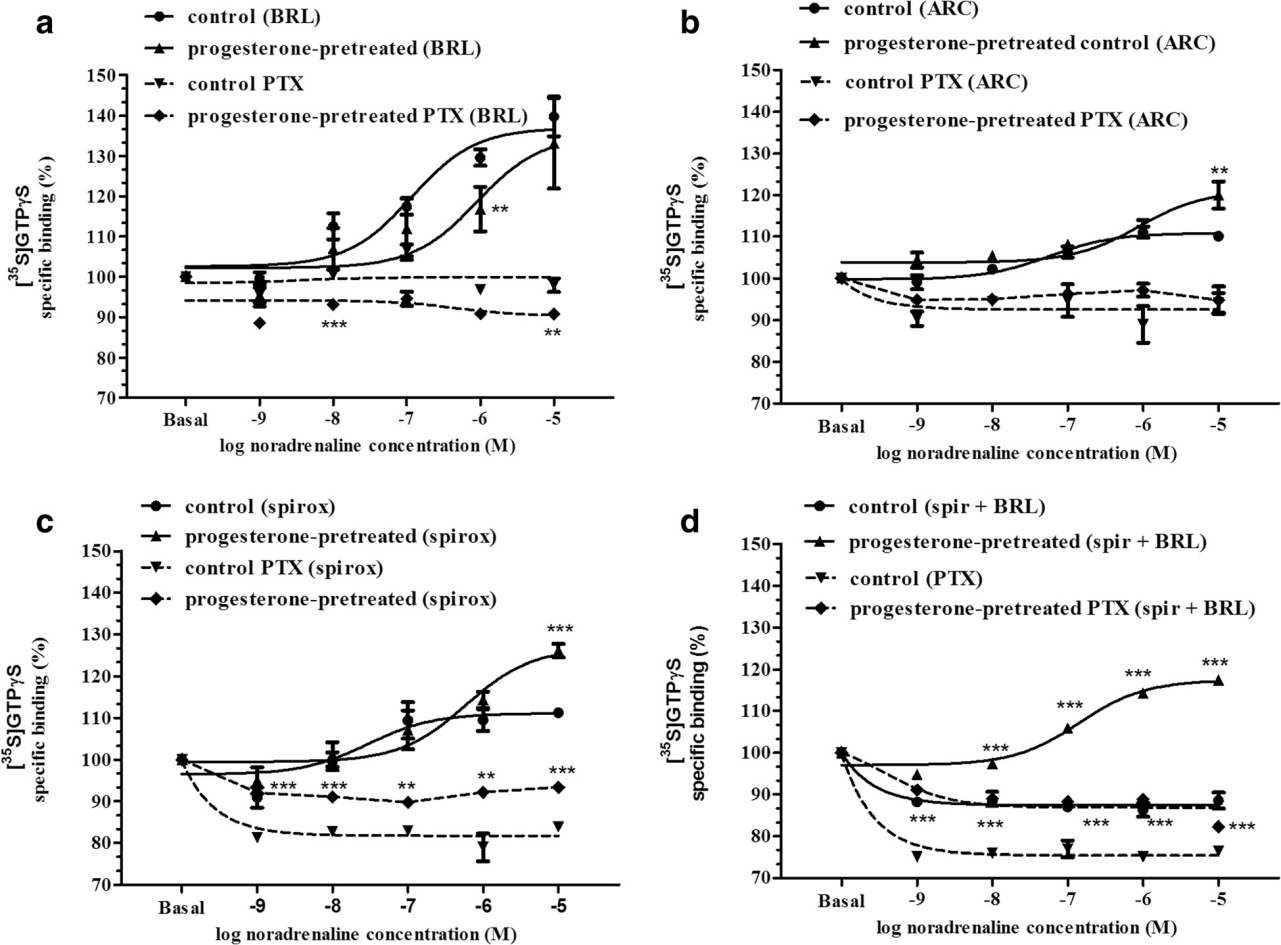
Changes induced by various concentrations of noradrenaline in [^35^S]GTPγS binding in the presence of the subtype-selective α_2A_-adrenergic receptor antagonist BRL 44408 (**a**), the α_2B/C_-adrenergic receptor antagonist ARC 239 (**b**), the α_2C_-adrenergic receptor antagonist spiroxatrine (**c**) and the spiroxatrine + BRL 44408 combination (**d**) following pretreatment with progesterone. In all cases, the β-adrenergic receptors and the α_1_-adrenergic receptors were inhibited by propranolol and doxazosin. Basal refers to the level of [^35^S]GTPγS binding without substance. The statistical analyses were carried out with the ANOVA Dunnett test. **p < 0.01; ***p < 0.001

In the presence of BRL 44408, (−)-noradrenaline increased the [^35^S]GTPγS binding, which was slightly decreased after progesterone pretreatment (*p* < 0.01). In the presence of pertussis toxin, the [^35^S]GTPγS binding-stimulating effect of (−)-noradrenaline ceased, and it was decreased further after progesterone pretreatment (*p* < 0.01) (Fig. 5a).

In the presence of ARC 239, (−)-noradrenaline moderately increased the [^35^S]GTPγS binding and it was more elevated after progesterone pretreatment (*p* < 0.01). In the presence of pertussis toxin, the [^35^S]GTPγS binding-stimulating effect of (−)-noradrenaline ceased, which was not modified even by progesterone pretreatment (Fig. 5b).

In the presence of spiroxatrine, (−)-noradrenaline slightly increased the [^35^S]GTPγS binding and it was more elevated (*p* < 0.001) after progesterone pretreatment. In the presence of pertussis toxin, however, (−)-noradrenaline elicited a decline in the [^35^S]GTPγS binding, to below the basal level from a concentration of 1 × 10^−9^ M. In the presence of pertussis toxin, progesterone pretreatment blocked the [^35^S]GTPγS binding-inhibitory effect of (−)-noradrenaline (Fig. 5c).

In the presence of the spiroxatrine + BRL 44408 combination, (−)-noradrenaline inhibited the [^35^S]GTPγS binding, but it was significantly increased after progesterone pretreatment (*p* < 0.001). In the presence of pertussis toxin, the spiroxatrine + BRL 44408 combination caused a dose-dependent inhibition in the [^35^S]GTPγS binding of (−)-noradrenaline, but the inhibition was reduced after progesterone pretreatment (Fig. 5d).

## Discussion

Since progesterone and the adrenergic system play major roles in the myometrial function during gestation, the main focus of our study was to clarify the effects of progesterone on the α_2_-AR subtypes in the late-pregnant uterine function in vitro. The α_2_-AR-selective action of (−)-noradrenaline was provided by the application of the α_1_-blocker doxazosin and the β-AR blocker propanolol. The applications of subtype-selective antagonists gave us the possibility to investigate the subtype-specific α_2_-AR responses to (−)-noradrenaline and to detect the modification induced by progesterone pretreatment. In an earlier study, we determined the subtype-selective α_2_-AR action of (−)-noradrenaline, and our present work therefore focused mainly on the influence of progesterone as a modifier of the α_2_-AR response [27].

Progesterone pretreatment increased the mRNA and protein expression of the myometrial α_2_-AR subtypes, but decreased the (−)-noradrenaline-evoked myometrial contraction through the α_2_-ARs, which was similar to our earlier findings with the α_1_-ARs [17].

In the isolated organ bath studies, progesterone pretreatment ceased the (−)-noradrenaline-evoked myometrial contraction through the α_2_-ARs, although it practically ceased the myometrial contracting effect of the (−)-noradrenaline through the α_2A_-ARs. Additionally, it abolished the myometrial contraction-increasing effect through the α_2B_-ARs, and reversed the myometrial contracting effect in the presence of BRL 44408 and in the presence of spiroxatrine. Since there are no available α_2A/B_-AR blockers to produce only α_2C_-AR stimulation, we can only presume that progesterone maintained the myometrial relaxing effect through the increased number and function of α_2C_-ARs.

To find an explanation of the weaker myometrial contractions via the α_2B_-AR subtype after progesterone pretreatment, we measured the myometrial cAMP level, since the changes in the cAMP level are involved in the myometrial effect of the α_2_-ARs. Progesterone pretreatment increased the myometrial cAMP level, which additionally proves the decreased myometrial contracting effect of (−)-noradrenaline through the α_2_-ARs. It did not alter the cAMP level through the α_2A_-ARs, which is in harmony with the result of the isolated organ bath studies that (−)-noradrenaline did not influence the myometrial contractions via these receptors after progesterone pretreatment. However, it increased the myometrial cAMP level through the α_2B_-ARs, which can explain the weaker myometrium-contracting effect of (−)-noradrenaline in the presence of BRL 44408 (stimulation via α_2B_- and α_2C_-ARs), spiroxatrine (stimulation via α_2A_- and α_2B_-ARs) and the spiroxatrine + BRL 44408 combination (stimulation via α_2B_-AR).

The literature indicates that the G_i_/G_s_-activating property of α_2_-AR in rats changes during gestation, resulting in differences in the regulation of myometrial adenylyl cyclase activity at mid-pregnancy versus term [32]. Moreover, progesterone induces a change in the G_q_/G_i_-activating property of α_1AD_-AR in rats [17]. We therefore measured whether progesterone can modify the myometrial [^35^S]GTPγS binding of the α_2_-AR subtypes in the presence of the G_i_ protein blocker pertussis toxin at the end of pregnancy. Progesterone did not modify the [^35^S]GTPγS binding of the α_2A_-ARs. However, via the α_2A_- and α_2B_-ARs (with spiroxatrine), progesterone reversed the effect of (−)-noradrenaline on the [^35^S]GTPγS binding in the presence of pertussis toxin and also increased the [^35^S]GTPγS binding-stimulating effect of (−)-noradrenaline. These findings indicate that progesterone modifies the coupling of α_2B_-ARs, but not the G protein binding of the α_2A_-ARs. To confirm this hypothesis, we measured the myometrial [^35^S]GTPγS binding of the α_2B_-AR subtype in the presence of the spiroxatrine + BRL 44408 combination. Progesterone reversed the effect of (−)-noradrenaline on [^35^S]GTPγS binding in the presence of pertussis toxin and also reversed the [^35^S]GTPγS binding-stimulating effect of (−)-noradrenaline. This result suggests that, in the presence of predominance of progesterone, the α_2B_-ARs are coupled, at least partially, to G_s_ protein, which leads to the activation of adenylyl cyclase and decreases the (−)-noradrenaline-induced myometrial contraction via these receptors.

## Conclusions

We conclude that progesterone increases the expression of each α_2_-AR subtype, and reduces the (−)-noradrenaline-induced myometrial contractions via the totality of these receptors. Progesterone blocks the G-protein coupling and cAMP production via the α_2A_-ARs. In the case of the α_2C_-ARs, we presume that progesterone treatment mainly induces the activation of the βγ subunit of the G_i_ protein, eliciting an increase in the smooth muscle cAMP level [19]. In the case of the α_2B_-ARs, G_s_ coupling is a determining factor in the function of the receptors after progesterone treatment, which leads to an increased cAMP level and decreased myometrial contraction.

Since the myometrial sensitivity to progesterone decreases at term, we assume that these changes can lead to the increased myometrial contraction near term via the α_2_-ARs. We presume that the effects of α_2C_-AR agonists and α_2B_-AR antagonists in combination with progesterone may open up new targets for drugs against premature birth.

## Abbreviations

AR, Adrenergic receptor; cAMP, Cyclic adenosine monophosphate; EC_50_, Half of the maximum effect; E_max_, Maximum effect; G protein, Heterotrimeric guanine nucleotide binding regulatory protein; GTPγS, Guanosine-5′-O-(γ-thio)triphosphate; NA, Noradrenaline; PTX, Pertussis toxin; RT-PCR, Reverse transcriptase-polymerase chain reaction; s.c., Subcutaneous; Tris–HCl, Tris(hidroxymethyl)aminomethane

## References

[1] Kamel RM. The onset of human parturition. Arch Gynecol Obstet. 2010. doi:10.1007/s00404-010-1365-9. 10.1007/s00404-010-1365-9 20127346

[2] Blanks AM, Shmygol A, Thornton S. Preterm labour. Myometrial function in prematurity. Best Pract Res Clin Obstet Gynaecol. 2007. doi:10.1016/j.bpobgyn.2007.03.003. 10.1016/j.bpobgyn.2007.03.003 17446138

[3] Wray S. Insights from physiology into myometrial function and dysfunction. Exp Physiol. 2015. doi:10.1113/EP085131. 10.1113/EP085131 26289390

[4] Illanes SE, Pérez-Sepúlveda A, Rice GE, Mitchell MD. Preterm labour: association between labour physiology, tocolysis and prevention. Expert Opin Investig Drugs. 2014. doi:10.1517/13543784.2014.905541. 10.1517/13543784.2014.905541 24717074

[5] Maggio L, Rouse DJ. Progesterone. Clin Obstet Gynecol. 2014. doi:10.1097/GRF.0000000000000039. 10.1097/GRF.0000000000000039 24936913

[6] Norwitz ER, Bonney EA, Snegovskikh VV, Williams MA, Phillippe M, Park JS, et al. Molecular Regulation of Parturition: The Role of the Decidual Clock. Cold Spri Harb Perspe Med. 2015. doi:10.1101/cshperspect.a023143. 10.1101/cshperspect.a023143 PMC4632866 25918180

[7] Renthal NE, Williams KC, Montalbano AP, Chen CC, Gao L, Mendelson CR. Molecular Regulation of Parturition: A Myometrial Perspective. Cold Spring Harb Perspect Med. 2015;5(11). Medline: 26337112 doi: 10.1101/cshperspect.a023069. 10.1101/cshperspect.a023069 PMC4632865 26337112

[8] Micks E, Raglan GB, Schulkin J. Bridging progestogens in pregnancy and pregnancy prevention. Endocr Connect. 2015. doi:10.1530/EC-15-0093. 10.1530/EC-15-0093 PMC4653318 26581227

[9] Gáspár R, Hajagos-Tóth J. Calcium channel blockers as tocolytics: principles of their actions, adverse effects and therapeutic combinations. Pharm (Basel). 2013. doi:10.3390/ph6060689. 10.3390/ph6060689 PMC3816733 24276256

[10] Marshall JM (1981). Effects of ovarian steroids and pregnancy on adrenergic nerves of uterus and oviduct. Am J Physiol.

[11] Hajagos-Tóth J, Bóta J, Ducza E, Csányi A, Tiszai Z, Borsodi A, Samavati R, Benyhe S, Gáspár R (2016). The effects of estrogen on the α2- adrenergic receptor subtypes in ratuterine function in late pregnancy in vitro. CMJ.

[12] Roberts JM, Riemer RK, Bottari SP, Wu YY, Goldfien A (1989). Hormonal regulation myometrial adrenergic responses: the receptor and beyond. J Dev Physiol.

[13] Dowell RT, Forsberg AL, Kauer CD (1994). Decreased ovarian blood flow may confound the tocolytic effect of ritodrine. Gynecol Obstet Invest.

[14] Engstrøm T, Vilhardt H, Bratholm P, Christensen NJ. Desensitization of beta2-adrenoceptor function in non-pregnant rat myometrium is modulated by sex steroids. J Endocrinol. 2001. doi:10.1677/joe.0.1700147. 10.1677/joe.0.1700147 11431147

[15] Gáspár R, Ducza E, Mihályi A, Márki A, Kolarovszki-Sipiczki Z, Páldy E, et al. Pregnancy-induced decrease in the relaxant effect of terbutaline in the late-pregnant rat myometrium: role of G-protein activation and progesterone. Reprod. 2005. doi:10.1530/rep.1.00490. 10.1530/rep.1.00490 15985637

[16] Gálik M, Gáspár R, Kolarovszki-Sipiczki Z, Falkay G. Gestagen treatment enhances the tocolytic effect of salmeterol in hormone-induced preterm labor in the rat in vivo. Am J Obstet Gynecol. 2008. doi:10.1016/j.ajog.2007.09.027. 10.1016/j.ajog.2007.09.027 18313455

[17] Bóta J, Hajagos-Tóth J, Ducza E, Samavati R, Borsodi A, Benyhe S, et al. The effects of female sexual hormones on the expression and function of α1A- and α1D-adrenoceptor subtypes in the late-pregnant rat myometrium. Eur J Pharmacol. 2015. doi:10.1016/j.ejphar.2015.11.015. 10.1016/j.ejphar.2015.11.015 26593425

[18] Xie N, Liu L, Li Y, Yu C, Lam S, Shynlova O, et al. Expression and function of myometrial PSF suggest a role in progesterone withdrawal and the initiation of labor. Mol Endocrinol. 2012. doi:10.1210/me.2012-1088. 10.1210/me.2012-1088 PMC5416987 22669741

[19] Zhou XB, Wang GX, Huneke B, Wieland T, Korth M. Pregnancy switches adrenergic signal transduction in rat and human uterine myocytes as probed by BKCa channel activity. J Physiol. 2000. doi:10.1111/j.1469-7793.2000.t01-1-00339.x. 10.1111/j.1469-7793.2000.t01-1-00339.x PMC2269869 10766916

[20] Knaus AE, Muthig V, Schickinger S, Moura E, Beetz N, Gilsbach R, et al. Alpha2-adrenoceptor subtypes--unexpected functions for receptors and ligands derived from gene-targeted mouse models. Neuroch Int. 2007. doi:10.1016/j.neuint.2007.06.036. 10.1016/j.neuint.2007.06.036 17664025

[21] Civantos Calzada B, Aleixandre de Artiñano A. α-adrenoceptor subtypes. Pharmacol Res. 2001. doi:10.1006/phrs.2001.0857. 10.1006/phrs.2001.0857 11529686

[22] Karim F, Roerig SC. Differential effects of antisense oligodeoxynucleotides directed against Gzα and Goα on antinociception produced by spinal opioid and α_2_ adrenergic receptor agonists. Pain. 2000. doi:10.1016/S0304-3959(00)00279-7. 10.1016/S0304-3959(00)00279-7 10924811

[23] Wang Q. α2-adrenergic receptors. Prim on the Auton Nerv Syst. 2012. doi:10.1016/B978-0-12-386525-0.00010-X.

[24] Offermanns S. G-proteins as transducers in transmembrane signalling. Prog Biophys Mol Biol. 2003. doi:10.1016/S0079-6107(03)00052-X. 10.1016/s0079-6107(03)00052-x 12865075

[25] Gyires K, Zádori ZS, Török T, Mátyus P. α_2_-adrenoceptor subtypes-mediated physiological, pharmacological actions. Neurochem Int. 2009. doi:10.1016/j.neuint.2009.05.014. 10.1016/j.neuint.2009.05.014 19477210

[26] Bouet-Alard R, Mhaouty-Kodja S, Limon-Boulez I, Coudouel N, Maltier JP, Legrand C. Heterogeneity of alpha 2-adrenoceptors in human and rat myometrium and differential expression during pregnancy. Br J Pharmacol. 1997. doi:10.1038/sj.bjp.0701555. 10.1038/sj.bjp.0701555 PMC1565112 9422821

[27] Gáspár R, Gál A, Gálik M, Ducza E, Minorics R, Kolarovszki-Sipiczki Z, et al. Different roles of alpha2-adrenoceptor subtypes in non-pregnant and late-pregnant uterine contractility in vitro in the rat. Neurochem Int. 2007. doi:10.1016/j.neuint.2007.06.029. 10.1016/j.neuint.2007.06.029 17664026

[28] Hajagos-Tóth J, Falkay G, Gáspár R. Modification of the effect of nifedipine in the pregnant rat myometrium: the influence of progesterone and terbutaline. Life Sci. 2009. doi:10.1016/j.lfs.2009.08.008. 10.1016/j.lfs.2009.08.008 19703476

[29] Chomczynski P, Sacchi N. Single-step method of RNA isolation by acid guanidinium thiocyanate-phenol-chloroform extraction. Anal Biochem. 1987. doi:10.1016/0003-2697(87)90021-2. 10.1006/abio.1987.9999 2440339

[30] Hajagos-Tóth J, Hódi Á, Seres AB, Gáspár R. Effects of d- and l-limonene on the pregnant rat myometrium in vitro. Croat Med J. 2015. doi:10.3325/cmj.2015.56.431. 10.3325/cmj.2015.56.431 PMC4655928 26526880

[31] Zádor F, Kocsis D, Borsodi A, Benyhe S. Micromolar concentrations of rimonabant directly inhibits delta opioid receptor specific ligand binding and agonist-induced G-protein activity. Neurochem Int. 2014. doi:10.1016/j.neuint.2013.12.005. 10.1016/j.neuint.2013.12.005 24508403

[32] Mhaouty S, Cohen-Tannoudji J, Bouet-Alard R, Limon-Boulez I, Maltier JP, Legrand C. Characteristics of the alpha 2/beta 2-adrenergic receptor-coupled adenylyl cyclase system in rat myometrium during pregnancy. J Biol Chem. 1995. doi:10.1074/jbc.270.18.11012. 10.1074/jbc.270.18.11012 7738044

